# Ecdysteroid UDP-Glucosyltransferase Expression in *Beauveria bassiana* Increases Its Pathogenicity against Early Instar Silkworm Larvae

**DOI:** 10.3390/jof9100987

**Published:** 2023-10-04

**Authors:** Xueqin Mao, Dongxu Xing, Die Liu, Haoran Xu, Luyu Hou, Ping Lin, Qingyou Xia, Ying Lin, Guanwang Shen

**Affiliations:** 1 Integrative Science Center of Germplasm Creation in Western China (Chongqing) Science City, Biological Science Research Center, Southwest University, Chongqing 400716, China; 2 Sericultural & Agri-Food Research Institute Guangdong Academy of Agricultural Sciences, /Key Laboratory of Functional Foods, Ministry of Agriculture and Rural Affairs, Guangdong Key Laboratory of Agricultural Products Processing, Guangzhou 510610, China

**Keywords:** fungal transgenic, 20E, deactivation, pathogenicity

## Abstract

*Beauveria bassiana (B. bassiana*) is a broad-spectrum entomopathogenic fungus that can control pests in agriculture and forestry. In this study, encoding ecdysteroid uridine diphosphate glucosyltransferase gene (*egt*) was successfully screened in *B. bassiana* on the medium containing 500μg/mL G418 sulfate solution through the protoplast transformation method. This enzyme has the function of 20E (20-hydroxyecdysone) inactivation, thus increasing the mortality of the early instar larvae infected with *B. bassiana*. In this study, we transformed *B. bassiana* with the *egt* gene, which deactivates 20-hydroxyecdysone, a key hormone in insect development. The results showed that transgenic *B. bassiana* killed more silkworms of the 2nd instar larvae than the wild-type with a shorter LT50 time, which was reduced by approximately 20% (day 1 of the 2nd instar silkworm infection of *B. bassiana*) and 26.4% (day 2 of the 2nd instar silkworm infection of *B. bassiana*) compared to the wild-type, and also showed a higher mortality number before molting. The transgenic *B. bassiana* had a higher coverage of the body surface of silkworms compared to the wild type on the 3rd instar. In summary, improving entomopathogenic fungi using biological methods such as genetic engineering is feasible.

## 1. Introduction

The increasing environmental pollution caused by chemical pesticides, the higher resistance of pests to pesticides, and the potential harm to human health caused by pesticides have raised concerns regarding the use of chemical pesticides. Thus, biological control is receiving more attention. Entomopathogenic fungi is one of the major factors in controlling insect populations in nature and is an important tool in the biological control of pests. *B. bassiana* is the most common and widely used environmentally-friendly fungus in various insects in nature [1,2]. It is the most common and widely used fungal insecticide [3]. However, the pathogenicity of different strains in the host is different, and pathogenicity is a comprehensive effect determined by the multiple characteristics of the strain. Compared to chemical pesticides, its slow efficacy and inconsistent control results are the main limitations of its large-scale application due to the fungi’s long growth and infection time [4,5]. Genetic modification of fungi using molecular biology techniques offers a faster and broader approach. Previous studies have demonstrated the increased expression of certain genes [6], such as protease Pr1A [4], CDEP1 [5], and chitinase CHIT1 [6] of *B. bassiana*, which are associated with the lethality of host infection. Some studies have also introduced exogenous toxin genes, such as the scorpion neurotoxin peptide AaIT1 [7,8] and the insecticidal proteins Vip3A [9] and Cry1Ac [10] from *Bacillus thuringiensis,* into *B. bassiana*, aiming to enhance its lethality to the host after infection.

*Beauveria bassiana* can infect more than 700 species of insects, including Lepidoptera, Hymenoptera, Coleoptera and 15 other orders, 149 families, 521 genera, and more than 10 species of ticks and mites in at least six families and seven genera [7]. As an important entomopathogenic fungus, *B. bassiana* is easy to cultivate and environmentally friendly to humans and animals. It has broad application prospects as an insecticide that biologically controls pests [1,8].

The fungal infection process in a host involves various steps, including host recognition, mechanical damage, nutrient competition, metabolic interference, toxin secretion, and tissue destruction in the host [9]. In particular, a certain amount of time is required for fungal conidia to adhere to the surface of the host insect, germinate, and penetrate the epidermis [10,11,12]. Most insects molt during their feeding period, and some insect instars have relatively short durations. Fungal infections may be eliminated before they invade the insect body, leading to potential failure [13]. Furthermore, although older instar insects have longer intervals between molts, the fungal infection must overcome the host’s immune system [14]. The fungal infections grow mycelia in the insect hemocoel, proliferate extensively, and secrete toxins using nutrients in the hemolymph, ultimately leading to the host insect’s death [15]. This process also requires a certain amount of time, during which the infected insects continue to feed. As a result, these pests can cause significant damage before their death following fungal infection. In conclusion, the fungus’s relatively long infection and mortality times on the host insect contribute to the relatively poor efficacy of fungal biocontrol agents. This is currently a major factor limiting the large-scale application of fungal insecticides. The second instar silkworm, *Bombyx mori*, larvae infected with *B bassiana*, molts after about 48 h of growth and development [16]. Under normal circumstances, it is difficult for conventional strains of *B. bassiana* to infect the second instar larvae of silkworms on a large scale. In contrast, the last instar larvae of the silkworms have a relatively longer duration. However, from infection with *B. bassiana* to death, these larvae continue to feed for at least 5 days [17].

Ecdysteroid UDP glucosyltransferase (EGT) is a protein encoded by baculovirus [18,19]. EGT secreted by Autograph californica nucleopolyhedral virus (AcMNPV) can transfer UDP-glucoside to the hydroxyl group of the 22nd carbon atom of 20E in vitro, forming 20E-22-β-D-glucopyranoside without 20E activity [20,21,22,23,24,25]. When insects are infected by this virus, the secreted EGT can deactivate 20E, thereby hindering the normal molting of insects [20,26] We previously found that the expression of EGT in the last instar silkworm led to a decrease in the content of active 20E in the hemolymph of the last instar silkworm, which made the silkworm unable to pupate after spinning [27]. This study aimed to integrate *egt* into the genome of *B. bassiana*, which was selected as the host to explore the function of endowing *B. bassiana* to deactivate 20E, increase the mortality of young larvae infected with *B. bassiana*, and, to a certain extent, compensate for the application limitations of *B. bassiana* in pest biological control due to its long pathogenic cycle.

## 2. Materials and Methods

### 2.1. Screening of Antibiotics

The *B. bassiana* strain CMCC(B)G1-180910 was preserved and provided by our research group. Experimental silkworms were provided by the Biological Science Research Center of Southwest University (Chongqing, China). Three gradients of antibiotic solutions–Hygromycin B (Sheng gong, Shanghai, China), Zeocin (Sheng gong, Shanghai, China), and G418 sulfate solution (Sheng gong, Shanghai, China) were prepared at 50, 100, and 200 μg/mL concentrations, respectively. A potato dextrose agar (PDA) medium containing the corresponding antibiotic concentrations was prepared, and a control group without antibiotics was included. Using sterilized clean tweezers, we gently removed mycelium from preserved *B. bassiana* strains and inoculated them onto the PDA medium to allow the white strain to grow in medium free of bacterial or other fungal contamination. *B. bassiana* was purified by single-spore culture. First, a few fresh conidia and hyphae were dipped by a sterile cotton swab, added to sterilized ddH_2_O, mixed well, and then passed through a 40 μm filter to remove mycelium. In this way, the conidia’ suspension was prepared. Next, 1 μL was sucked for observation under a microscope and diluted with sterile water until the spore concentration was 1 μL, comprising only a single spore. This spore was inoculated and cultured in a petri dish. The purified *B. bassiana* strain was inoculated onto agar plates using sterile cotton swabs to ensure uniform distribution. It was then moderately dipped with sterile cotton swabs and evenly applied onto the PDA plate until no white conidia or mycelium were visible. The plates were inverted and cultured at 26 °C with a relative humidity (RH) of at least 90%. Suitable antibiotics and their concentrations were determined based on the growth of the *B. bassiana* colonies. If antibiotics had no inhibitory effect on the growth of *B. bassiana*, the PDA plate coated with the strain would be covered with white strain, and if they did have an inhibitory effect, it would not grow.

### 2.2. Construction of Transgenic Fungal Expression Vector

After optimizing the target gene containing EGT by using the preference codon of *B. bassiana* provided in the NCBI database, the Mcl1 signal peptide (SP, ATGCGTGAGCTCTCCTCCGTTCTCGCTCTCTCCGGTCTCCTCGCTCTCGCTTCCGCT) and 6×His tag was added to the 5′ end of the *egt* gene. The linker (GGCGGCGGTGGTTCT) and nucleic acid sequence of green fluorescent protein (GFP) were added at the 3′ end. A nucleic acid sequence containing sp-6×His-EGT-linker-GFP with *Xho* I and *Kpn* I sites was synthesized by Takara Bio Corporation and cloned into the pMD19-T plasmid to generate pMD19-T [sp-6×His-EGT-linker-GFP]. pMD19-T [sp-6×His-EGT-linker-GFP] and the fungal expression vector pG418, containing the G418 resistance gene, were digested with *Xho* I and *Kpn* I, respectively. The recovered sp-6×His-EGT-linker-GFP was ligated into a transgenic vector. The *Xho* I-*Kpn* I fragment from pMD19-T [sp-6×His-EGT-linker-GFP] was excised and inserted into the *Xho* I-*Kpn* Ⅰ site of pG418 to generate pG418 [sp-6×His-EGT-linker-GFP]. The pG418 fungal expression vector was kindly provided by Professor Yi Zou of the College of Pharmaceutical Sciences at Southwest University (Chongqing, China).

### 2.3. Construction and Selective Culture of Transgenic B. bassiana

The protoplast transformation method has been widely applied in the genetic transformation of fungi. Transgenic *B. bassiana* were generated as previously described [28], the details of which are following. *B. bassiana* colonies grown on PDA medium for 14 days were collected using a sterile 20% glycerol solution and the mycelia were filtered to obtain a spore suspension. The spore suspension was cultured at 25 °C, 220 *rpm* shake culture for 12 h, and obtained 70~80% of the spore germinated buds (the length of the buds was approximately 3~4 times the diameter of the conidia). The germinated spore buds in a 50 mL culture medium were collected and washed twice using 15 mL OM buffer (The OM buffer, including 1.2 3M MgSO_4_·7H_2_O and 10 mM Na–Phosphate buffer, was filter sterilized and stored at 4 °C. and the pH was adjusted to 5.8 using 1 M Na_2_HPO_4_) via centrifugation at 4 °C and 4000× *g* for 5 min. The 10 mM Na–Phosphate buffer was prepared from a 2 M NaPB stock solution containing 90.9 g of Na_2_HPO_4_ and 163.4 g of NaH_2_PO_4_ per liter, with a pH of 6.5. Next, 10 mL mixed enzyme solution of 0.03 g Lysing Enzymes from *Trichoderma harzianum* (Sigma-Aldrich, St. Louis, MO, USA) and 0.02 g Yatalase (Takara Bio, Otsu, Japan) dissolved in 10 mL OM buffer were added to digest the new buds. Protoplasts of *B. bassiana* were obtained via culturing at 28 °C and 80 *rpm* shake culture for 13 h. Then, 10 mL of trapping buffer (0.6 M sorbitol; 0.1 M Tris-HCl, pH 7.0; autoclaved and stored at 4 °C) were added and protoplast layers were collected by centrifugation at 4 °C and 4000× *g* for 20 min. Then, after being added to 10 mL STC buffer (1.2 M sorbitol; 10 mM CaCl_2_·2H_2_O; 10 mM Tris-HCl, pH 7.5; autoclaved and stored at 4 °C), the precipitate was obtained by centrifugation at 4 °C and 4000× *g* for 8 min and resuspended in 300 μL STC buffer. The 10 μL transgenic fungal expression vector pG418-sp-His-EGT-GFP ultrapure plasmid was mixed with 100 μL protoplasts and allowed to stand on ice for 40 min. Then, 600 μL PEG solution (60% PEG 4000; 50 mM CaCl_2_; 50 mM Tris-HCl, pH 7.5; autoclaved and stored at 25 °C) was added and mixed evenly, standing at 25 °C for 30 min. The mixture was added to an SD-PDA (PDA with 21.86% sorbitol) plate containing 500 μg/mL G418 sulfate solution prepared in advance, and the mixture was manually rolled and spread. Under 26 °C, RH ≥ 90%, the mixture was cultured for 1 day and then inverted for 4 days. A white colony was selected and continuously cultured on PDA plate containing 500 μg/mL G418 sulfate solution, purer transgenic strains were obtained after 20 generations of single-spore cultured on PDA plates containing 500 μg/mL G418 sulfate solution.

### 2.4. Identification of Transgenic B. bassiana

The target strain was scraped with a sterile cotton swab and inoculated into a sterile test tube containing 8 mL of PDB medium. After shaking the culture at 26 °C and 200 *rpm* shake culture for 3 days, the precipitate was collected via centrifugation at 4 °C and 12,000× *g* for 3 min. After adding 700 μL CTAB buffer (2% CTAB; 8.182% NaCl; 0.1 M Tris-HCl, pH 8.0; 0.02 M EDTA, pH 8.0) and transferring to 1.5 mL sterile centrifuge tube, the mycelium fluid was quickly frozen with liquid nitrogen for 2 min, followed by a 65 °C water bath for 2 min, and repeatedly freeze-thawed five times. Then, 600 μL of the liquid was piped into a new 1.5 mL sterile centrifuge tube and added to 600 μL phenol: chloroform: isoamyl alcohol (25:24:1, *V/V/V*). The 500-μL supernate was obtained via centrifugation at 13,000× *g* for 10 min and then 500 μL phenol: chloroform: isoamyl alcohol (25:24:1, *V/V/V*) was added. After, 400 μL of supernate was obtained via centrifugation at 13,000× *g* for 10 min and added to 800 μL precooled isopropanol, then allowed to stand at −20 °C for 40 min. After centrifugation at 13,000× *g* for 5 min, the precipitate was washed using 600 μL 70% ethanol, dried at room temperature, and dissolved in sterile water sequentially. The total RNA was extracted using the RNA extraction kit SteadyPure Universal RNA Extraction Kit (Accurate Biology, Changsha, Hunan, China), then first-strand cDNA was generated using reverse transcription kit NovoScript^®^ Plus All-in-one 1st Strand cDNA Synthesis SuperMix (gDNA Purge) (Novoprotein, Suzhou, China), according to the manufacturer’s protocol.

Polymerase chain reaction (PCR) for *egt* fragment from *B. bassiana* genomic DNA. The PCR profile was used to amplify target sequences: 94 °C for 5 min; 30 cycles of 94 °C for 30 s, 60 °C for 30 s and 72 °C for 1 min; 72 °C for 10 min. PCR for the full-length sp-His-EGT-GFP from *B. bassiana* cDNA was used to amplify target sequences: 94 °C for 5 min; 30 cycles of 94 °C for 30 s, 58 °C for 30 s and 72 °C for 2 min 40 s; 72 °C for 10 min. 18S rRNA was used as the control. The PCR profile was 94 °C for 5 min; 30 cycles of 94 °C for 30 s, 58 °C for 30 s and 72 °C for 20 s; 72 °C for 10 min. Electrophoresis confirmed products on a 1.2% (*w/v*) agarose gel. The primers involved in PCR are shown in Table S1.

### 2.5. Validity Detection of Transgenic Beauveria bassiana

The wild-type and transgenic *B. bassiana* were inoculated in a PDA medium containing effective antibiotics, 26 °C, RH ≥ 90%, and cultured for 14 days. Conidia were scraped and dispersed in 0.05% Tween-80 solution to obtain a spore suspension with 1 × 10^7^ conidia/mL concentration. Silkworm larvae were selected as the test insects on the first day of the second instar, the second day of the second instar, and the first day of the third instar. Each group comprised 300 silkworms of similar size. The spore suspension was evenly sprayed on small silkworms to observe the moisture on the body surface. A 0.05% Tween-80 solution without spore was used as a negative control. After 30 min of inoculation, mulberry leaves were fed to the silkworms, the temperature was raised to 26 °C, and the RH ≥ 90%. Silkworm excrement was cleaned, and fresh mulberry leaves were replaced daily. The death of each treatment was observed and recorded at 12 h intervals, and the median time to death of the test insects was counted prior to the death of wild-type and transgenic *B. bassiana*-infected silkworm. The transgenic *B. bassiana* treatment group was the experimental group, and the wild-type *B. bassiana* treatment group was the control group. Fresh *B. bassiana* with white villous hyphae and powdery spore on silkworm carcasses were selected, and the hyphae and conidia were collected. The genome and RNA were extracted and detected by PCR, as described above.

### 2.6. Data Collection and Visualization

Microsoft Excel was used to organize the raw data and graph the survival curves and mortality rates.

## 3. Results

### 3.1. Screening of Antibiotics and Determination of Culture Conditions

**Figure 1:**
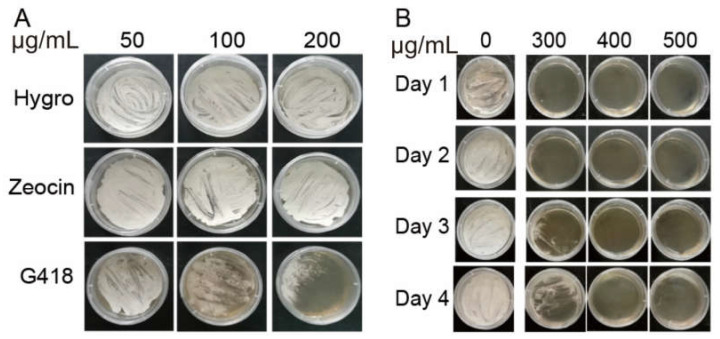
Screening of antibiotics and culture conditions of *Beauveria bassiana*. (**A**) The growth of *B. bassiana* on potato dextrose agar (PDA) solid medium with different concentrations of Hygromycin B, Zeocin, and G418 sulfate solution; (**B**) The growth of *B. bassiana* on PDA solid medium supplemented with different concentrations of G418 sulfate solution.

To screen and culture positive transgenic *B. bassiana*, sensitive antibiotics and the optimal concentration of *B. bassiana* were screened using the PDA medium. First, the activated *B. bassiana* conidia were inoculated on PDA solid medium with 50, 100, and 200 μg/mL of Hygromycin B, Zeocin, and G418 sulfate solution, respectively. After two days of culture, *B. bassiana* was grown on a medium containing 200 μg/mL Hygromycin B and Zeocin. The growth of *B. bassiana* was inhibited in the medium containing the G418 sulfate solution, and the higher the concentration, the more pronounced the inhibition (Figure 1A). Furthermore, *B. bassiana* was cultured on PDA solid medium containing 300 μg/mL, 400 μg/mL, and 500 μg/mL G418 sulfate solution for 4 days; the 500 μg/mL G418 sulfate solution could inhibit the growth of *B. bassiana* on PDA medium (Figure 1B). The above results showed that within 4 d, PDA medium containing 500 μg/mL G418 sulfate solution could be used as a screening medium for the genetic transformation of *B. bassiana* and positive transgenic strains of transformed offspring.

### 3.2. Generation of Transgenic Beauveria bassiana

**Figure 2:**
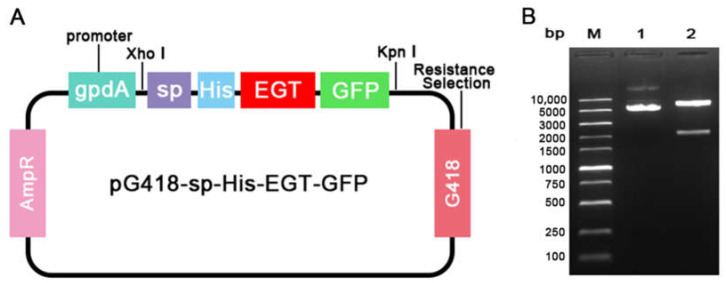
Construction of transgenic fungal expression vector. (**A**) Schematic illustration of the transgenic fungal expression vector pG418-sp-His-EGT-GFP; (**B**) The detection of recombinant fungal expression vector by double enzymes digestion.

The recombinant fungal expression vector pG418-sp-His-EGT-GFP driven by the gpdA constitutive promoter was digested with the restriction enzymes *Xho* I and *Kpn* I (Figure 2A). The size of the vector was 6365 bp, whereas the size of the target fragment was 2286 bp, consistent with the expected results (Figure 2B). This confirmed the successful construction of the transgenic fungal expression vector pG418-sp-His-EGT-GFP.

**Figure 3:**
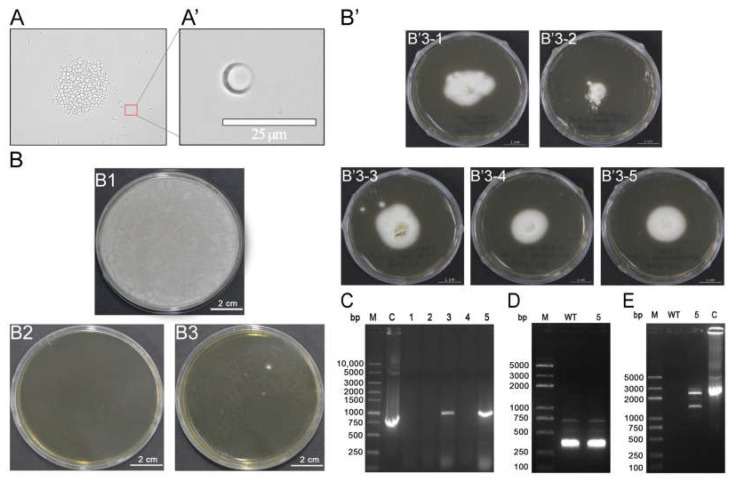
Generation and identification of transgenic *Beauveria bassiana*. (**A**) The protoplasts obtained from *B. bassiana*; (**A’**) Enlarged view of individual protoplast; (**B**) Screening of positive transgenic *B. bassiana*, (**B1**): the culture media without antibiotic selection, (**B2**): the culture media containing 500 μg/mL G418 sulfate solution without the plasmid, (**B3**): the culture media containing 500 μg/mL G418 sulfate solution with plasmid transfer; (**B’**) Cultivation of transformants, B’3 (1–5): five transformed colonies selected from B3, which is cultured for 3 days on PDA plates containing G418 sulphate; (**C**) 1–5: Gel electrophoresis of genomic PCR products of five transformants, C: PCR product of control plasmid pG418-sp-His-EGT-GFP; (**D**) Gel electrophoresis of 18S ribosomal RNA in transformant 5 RNA; (**E**) Gel electrophoresis of the target gene in transformant 5 RNA.

Using lysing enzymes from *T. harzianum* and Yatalase, the newly germinated hyphae of *B. bassiana* were enzymatically digested to obtain protoplasts. Successfully digested protoplasts appeared as transparent circular structures (Figure 3A). The protoplasts were then transformed with the pG418-sp-His-EGT-GFP highly purified plasmid and uniformly spread on the corresponding culture media. After 4 days of cultivation, the culture media without antibiotic selection showed the growth of white mycelia (Figure 3(B1)), whereas no colony growth was observed in the culture media containing 500 μg/mL G418 sulfate solution without the plasmid (Figure 3(B2)). However, on G418 sulfate-containing PDA plates with plasmid transfer, single white fungal colonies emerged (Figure 3(B3)), indicating that the antibiotic effectively inhibited the growth of non-transgenic *B. bassiana*. Five randomly selected single fungal colonies were cultivated on G418 sulfate-containing PDA plates for 3 days. The transformed colonies showed powdery white growth with a radially symmetrical pattern (Figure 3B’). The genomic DNA of these five transformants were extracted, and PCR analysis revealed the amplification of an *egt* fragment of approximately 1000 bp in transformant B’3–5 (Figure 3C). Furthermore, RNA extracted from transformant B’3-5 confirmed the presence of 18s rRNA (Figure 3D) and the successful amplification of a target gene of approximately 2300 bp (Figure 3E). These results demonstrated the successful generation of transgenic *B. bassiana* containing EGT.

### 3.3. Validity Detection of Transgenic Beauveria bassiana

**Figure 4:**
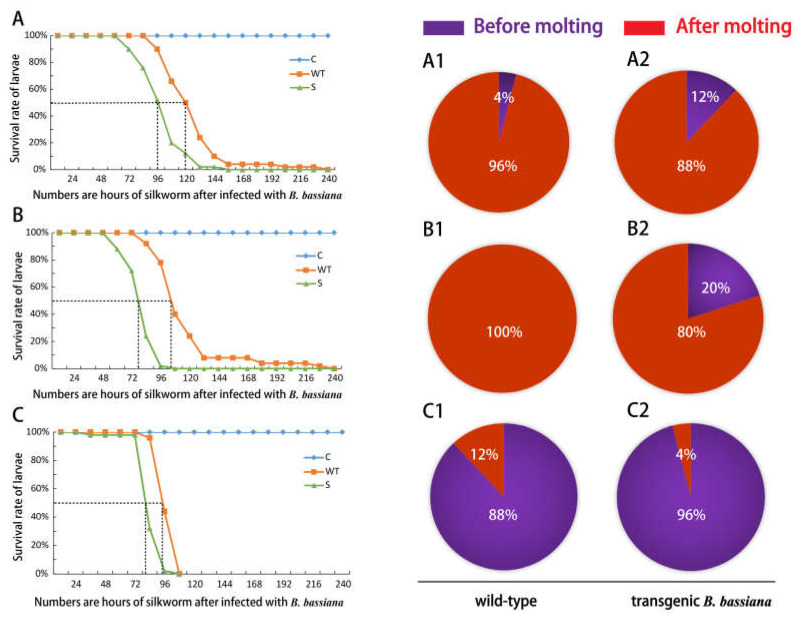
Validity detection of transgenic *Beauveria bassiana*. (**A**,**B**) The survival rate of silkworms inoculated with *B. bassiana* on the 1st/2nd day of the second instar, the ratio of death of silkworms before and after molting of the second instar inoculated with *B. bassiana* on the 1st day of the second instar of (**A1**,**A2**), and the ratio of death of silkworms before and after molting of the second instar of (**B1**,**B2**) inoculated with *B. bassiana* on the 2nd day of the second instar; (**C**) The survival rate of silkworms inoculated with *B. bassiana* on the first day of the third instar, the ratio of death of silkworms before and after molting of the third instar inoculated with *B. bassiana* on the first day of the third instar of (**C1**,**C2**). C: Negative control group treated with 0.05% Tween-80 solution; WT: control group treated with wild-type *B. bassiana*; S: Experimental group treated with transgenic *B. bassiana*.

The median lethal time (LT50) of transgenic *B. bassiana* infecting silkworm larvae on the 1st and 2nd day of the second instar was shortened by 24 h compared to that of the wild-type (Figure 4A,B), while the LT50 of the transgenic *B. bassiana* was only 12 h shorter than that of the wild-type on the first day of the third instar larvae (Figure 4C). These results showed that the pathogenicity time of transgenic *B. bassiana* was shortened, and the effect on second-instar silkworms was more obvious. On the other hand, EGT has the function of hindering insect molting to take into consideration. We also collected the number of deaths before and after the molting of silkworms infected with *B. bassiana*. Comparing the inoculation of silkworms with *B. bassiana* on the first day with the second day of second instar larva, the number of silkworms that died before molting infected by transgenic *B. bassiana* was three times that of the wild-type (Figure 4(A1,A2)). On the second day of inoculation wild-type *B. bassiana*, all the silkworms died after molting, while there was still 20% of silkworms that died before molting infected by transgenic *B. bassiana* (Figure 4(B1,B2)). Even if inoculation has long period molting intervals for third instar silkworms, the number of silkworms infected by transgenic *B. bassiana* (96%) died before molting was still greater than those inoculated with wild-type *B. bassiana* (88%) (Figure 4(C1,C2)). This confirms that the transgenic *B. bassiana* increases in the availability of killed silkworms that die before molting.

**Figure 5:**
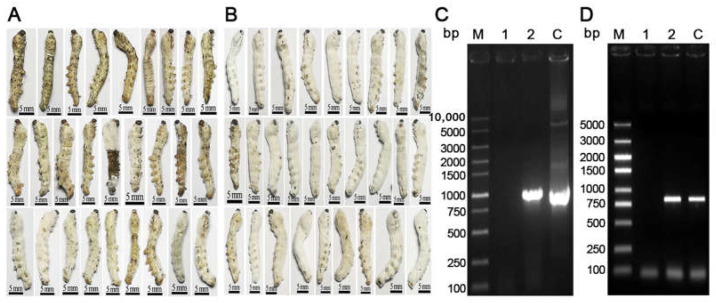
Pathogenicity analysis of transgenic *Beauveria bassiana*. (**A**) The phenotype of the third instar silkworms infected with wild-type *B. bassiana* after all death; (**B**) The phenotype of the third instar silkworms infected with transgenic *B. bassiana* at the same time; (**C**) Electrophoresis detection of PCR products of the target gene fragment in the genome of *B. bassiana*, 1: wild-type *B. bassiana* genome template, 2: transgenic *B. bassiana* genome template, C: control plasmid pG418-sp-His-EGT-GFP template; (**D**) Electrophoresis detection of PCR products of the target gene in *B. bassiana* RNA, 1: wild-type *B. bassiana* cDNA template, 2 transgenic *B. bassiana* cDNA template, C: control plasmid pG418-sp-His-EGT-GFP template.

Third-instar silkworms infected with *B. bassiana* died (108 h after inoculation), the phenotypes of 30 small silkworms were observed by photography. The proportion of small silkworms with mycelia on their body surface was approximately three times that of the control group. Some dead silkworms (Figure 5B) grew hyphae and conidia. Considering that the molting interval of the third instar silkworm (96 h) was longer than that of the second instar silkworm (48 h), *B. bassiana* could cause most silkworms to die before the third instar molting. Silkworms infected with transgenic *B. bassiana* exhibited greater ossification during the same period. The genome and RNA of *B. bassiana* from silkworm corpses were extracted for PCR detection. The results showed that the target gene fragment was successfully amplified in the experimental group with a size of approximately 1000 bp, consistent with the band size amplified from the control plasmid. In contrast, the control group was not amplified (Figure 5C). The difference observed in this study was due to the introduction of EGT into transgenic *B. bassiana*.

## 4. Discussion

It should be noted that there are multiple methods for the genetic manipulation of fungal species [29]. Currently, there is no universal transformation method applicable to all fungi, and specific transformation protocols must be designed for different species. Therefore, it is of great practical significance to continuously explore and discover more efficient and suitable transformation methods for *B. bassiana* to improve the strains and facilitate future applications. In this study, we chose the protoplast transformation method because it is simple, effective, and does not require expensive equipment. Using protoplasts as recipient cells, this method produces many transformants and easily obtains homozygous transformants. However, this method inevitably has disadvantages, such as a low frequency of positive transformant regeneration and a long purification period. Additionally, the selection of positive strains using this method is commonly achieved through antibiotic resistance; however, different fungi have varying sensitivities to different antibiotics. To obtain positive strains efficiently and accurately, further exploration of plasmid resistance selection, antibiotic concentrations, and fungal culture methods is needed. In this study, we found that a solution of G418 sulfate at a concentration of 500 μg/mL completely inhibited the growth and reproduction of *B. bassiana* within 4 days. Therefore, it was used as a screening marker for the genetic transformation of *B. bassiana* and as a selective pressure for offspring after transformation. These findings provide a reference for selecting *B. bassiana* strains carrying other genes. However, it is necessary to explore further whether other antibiotics can make *B. bassiana* more sensitive.

Many genes have been introduced into *B. bassiana* to enhance its pathogenicity. Fungal infections of insects first penetrate the exoskeleton of insects, which is mainly composed of chitin and proteins. During the process of fungal infection of insect body surfaces, enzymes such as protease (Pr1A) [30] and Pr1A like proteases CDEP1 [31], and chitinase CHIT1 [32], are synthesized to dissolve insect body walls and aid in fungal penetration [2,12]. Therefore, increasing the expression of such proteins in fungi through transgenic transformation is one of the means to enhance their pathogenicity. In addition, some non-fungal endogenous proteins that have toxic effects on insects can be used to enhance fungal virulence such as the crystal insecticidal proteins (Vip3A) and Cry1Ac of *B. thuringiensis*, which can dissolve insect midgut epithelial cells, leading to cell lysis [33]. Scorpion neurotoxic peptide (ATM1) can cause instant spastic paralysis in insects [34]. The main goal of this study is to address the issue of short interval between molting of the early instar insect larvae, which leads to shedding of epidermis of *B. bassiana*-infected larvae after molting. Shedding of epidermis significantly affects the success of *B. bassiana* infection. Shedding of epidermis significantly affects the success of *B. bassiana* infection. Therefore, we utilized the characteristic of EGT to deactivate 20E [21,25,27] and expressed EGT in *B. bassiana* to inhibit the molting hormone in insects, thus extending the interval between molting of early instar larvae to improve the success rate of *B. bassiana* infection. Due to cost and limitations in experimental facilities, the conclusions of this study have only been validated in the laboratory. To confirm the effectiveness of larger-scale applications or the establishment of a relevant technological systems, further verification is needed by expanding the sample population in different or the same varieties of silkworms.

Lepidoptera is the second largest order in the class Insecta, with the most agricultural and forestry pests. Silkworms are commonly used as model insects for research on lepidopteran [35,36] and fungal infections [37]. In this study, although the improved genetically modified *B. bassiana* in this study theoretically has broad-spectrum biological control against Lepidoptera pests, its true effect still needs to be verified in other insects. In addition, it is noted that although the molting of larvae from almost all insects requires 20E to participate [38], the molting interval of late-stage larvae is inherently long (around 10 days for the last instar silkworms). During this time, the advantages of our genetically modified *B. bassiana* strain compared to the wild-type strain may weaken or even disappear. Therefore, regardless of whether it is the endogenous protein of the fungus or the exogenous protein, the extent of improvement is limited by the expression level of the target protein and the duration of application. In the future, genes that can enhance fungal infection efficiency (such as CDEP1 and CHIT1), genes toxic to insects (such as Vip3A and Cry1Ac proteins), and genes affecting insect molting (EGT) can be simultaneously expressed in *B. bassiana* or mixed in different strains of *B. bassiana* containing these proteins to enhance the insecticidal activity of *B. bassiana* against harmful insects and ultimately expand the application of *B. bassiana* in biological control.

## 5. Conclusions

Based on the characteristic of insect molting, we focused on “giving” *B. bassiana* a new function—deactivating 20E. This successfully caused the death of second instar silkworm larvae, which are usually difficult to infect with *B. bassiana* because of their short molting period. This approach partially compensates for the limitations of the long pathogenic cycle of *B. bassiana* in the application of pest biological control and provides insights into the improvement of biocontrol fungi and their efficacy against other insects.

## Supplementary Materials

The following supporting information can be downloaded at: https://www.mdpi.com/article/10.3390/jof9100987/s1, Table S1: Primers used in the study.
